# Frailty and Pre‐Frailty in Patients With Lung Cancer and Its Association With Long‐Term MACCE: A Longitudinal Cohort Study

**DOI:** 10.1002/cam4.71458

**Published:** 2025-12-08

**Authors:** Fang Zhu, Qian Zhang, Hang Hao, Wenjie Cai, Qingquan Luo

**Affiliations:** ^1^ Department of Surgical Oncology Shanghai Lung Cancer Center, Shanghai Chest Hospital, Shanghai Jiao Tong University School of Medicine Shanghai China; ^2^ Bengbu Medical University Bengbu China; ^3^ Department of Epidemiology Johns Hopkins University Bloomberg School of Public Health Baltimore Maryland USA

**Keywords:** cardio‐oncology, frailty, long‐term outcome, lung cancer, MACCE, pre‐frailty

## Abstract

**Background:**

Lung cancer (LC) is a leading cause of morbidity and mortality worldwide. Cardiovascular disease is the primary cause of non‐cancer‐related death among cancer survivors. Frailty, characterized by a decline in physiological reserves, has been identified as a predictor of poor outcomes in cancer. However, the relationship between frailty, pre‐frailty, and long‐term cardiovascular outcomes in LC patients remains insufficiently explored.

**Methods:**

This retrospective analysis of a cohort study utilized prospectively collected data from the UK Biobank, with baseline assessment between 2006 and 2010 and follow‐up until October 31, 2022. Frailty was defined using the frailty phenotype according to five components (weight loss, exhaustion, low physical activity, slow gait speed, and low grip strength). Participants were categorized as non‐frail, pre‐frail or frail. The outcome was defined as major adverse cardiac and cerebrovascular events (MACCE). Cox proportional hazards models adjusted for confounders including age, sex, obesity, smoking status, socioeconomic status, diabetes, hypertension, COPD, and tumor type were employed to estimate hazard ratios (HR) for MACCE.

**Results:**

Of the 500,530 participants in this cohort, 6095 were diagnosed with LC after recruitment. Among LC patients, 43.79% were non‐frail, 48.20% pre‐frail, and 8.01% frail. Frail individuals had a significantly higher risk of MACCE (HR = 1.21, 95% CI: 1.07–1.38, *p* = 0.002) compared to non‐frail patients, while pre‐frail individuals also exhibited an elevated risk (HR = 1.10, 95% CI: 1.02–1.18, *p* = 0.010). Specific frailty components, particularly low physical activity and slow gait speed, were strongly associated with increased risks of both MACCE and all‐cause mortality. In contrast, low grip strength did not show a significant association with adverse outcomes.

**Conclusions:**

LC participants had a higher prevalence of pre‐frailty and frailty. The presence of frailty and pre‐frailty significantly increased the risk of MACCE in long‐term LC survivors. Notably, slow gait speed and low physical activity were strongly associated with MACCE compared to other frailty components.

**Abbreviations**
ANOVA: analysis of variance
BMI: body mass index
CHD: coronary heart disease
CI: confidence interval
COPD: chronic obstructive pulmonary disease
HF: heart failure
HR: hazard ratio
ICD‐9: international classification of diseases, 9th revision
ICD‐10: international classification of diseases, 10th revision
ICD‐O‐3: international classification of diseases for oncology, 3rd edition
IQR: interquartile range
LC: lung cancer
MACCE: major adverse cardiac and cerebrovascular events
MET: metabolic equivalent task
NSCLC: non‐small cell lung cancer
RF: respiratory failure
UK: United Kingdom
UK Biobank: United Kingdom Biobank
y: year

## 1 Introduction

Lung and bronchus (hereinafter lung) cancer represents a significant global disease burden, with an estimated 1.8 million deaths in 2020 [1, 2]. According to a population‐based registry, the prevalence of cardiovascular co‐morbidity among lung cancer (LC) patients is about twice as high as in the general population [3]. With improved cancer treatments and a rising survivorship population, which is estimated to be 26 million by 2040, [4] it is critical to manage cardiovascular comorbidities in patients with cancer, especially in the elderly individuals [5].

Frailty, an emerging public health concern worldwide paralleled with population aging, is characterized by a decline in functioning across multiple physiological systems, with a resultant increased susceptibility to stressors [6, 7]. Frailty is an important shared risk factor for both cancer and cardiovascular disease [8]. The prevalence of frailty in LC was45% [9]. In patients with LC, frailty was associated with a 3‐fold increased risk for mortality [9]. While the relationship between frailty and mortality in patients with cancer is well documented, the long‐term cardiovascular consequences, specifically the risk of major adverse cardiac and cerebrovascular events (MACCE), remain poorly understood in LC patients. Pre‐frailty is a condition characterized by the presence of some but not all features of frailty, representing an intermediate stage in the continuum from health to frailty [10]. Pre‐frailty is considered a critical stage for early intervention, as it is associated with an increased risk of progression to frailty and mortality, especially for older patients with cancer [11]. Moreover, the role of pre‐frailty as a precursor to frailty and its potential impact on cardiovascular risk in this population has yet to be explored in depth.

In this study, we utilized the UK Biobank, a large nationwide cohort study, to further investigate the association between frailty and pre‐frailty with long‐term MACCE in LC patients. By identifying frailty as a risk factor for MACCE in LC patients, this study has the potential to inform clinical practice, leading to improved risk stratification and better long‐term outcomes for LC patients at risk for cardiovascular events.

## 2 Methods

### 2.1 Study Group

**FIGURE 1:**
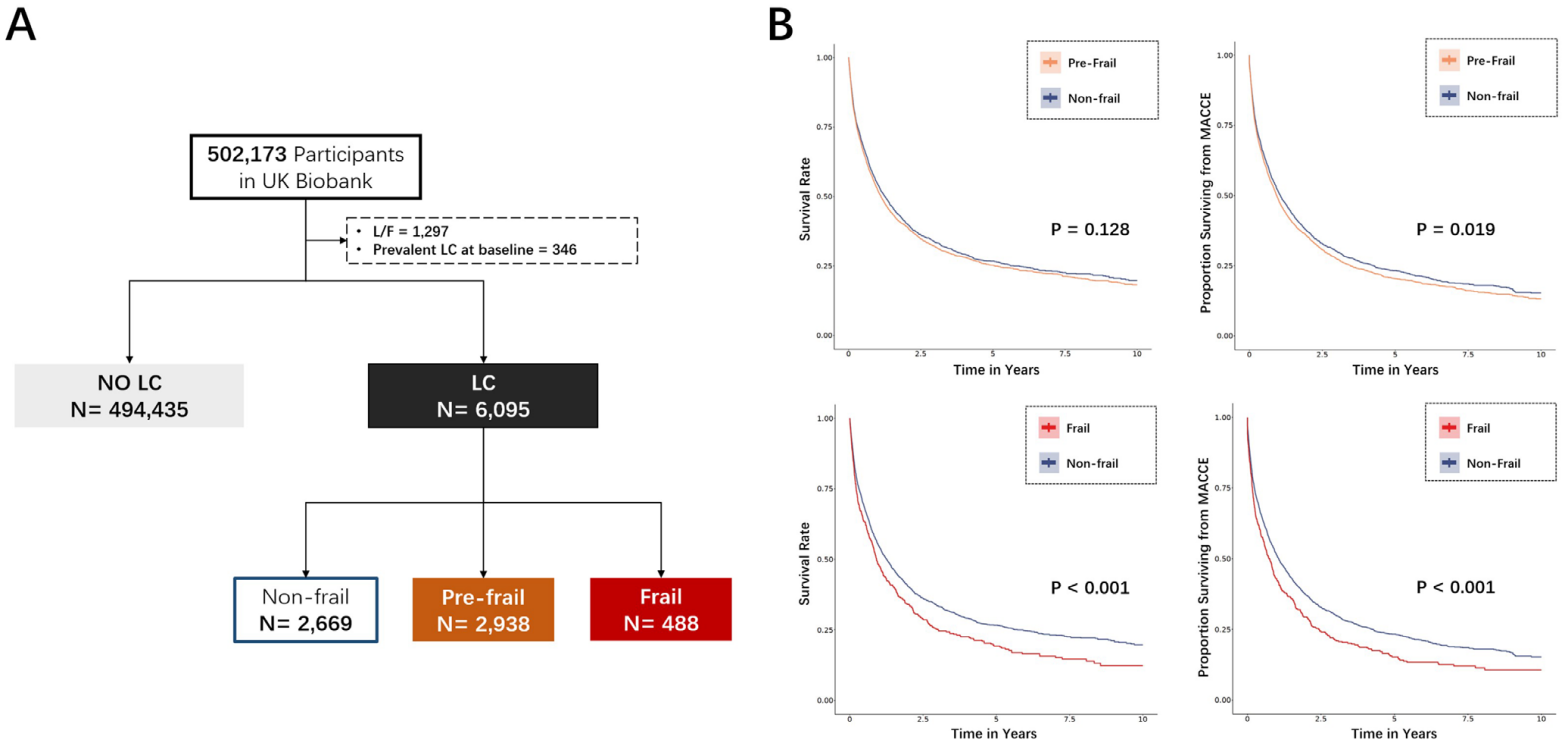
Flow chart of inclusion and Kaplan–Meier estimates of the probability of death and MACCE in LC participants without frailty, with pre‐frailty and frailty. (A) Flow chart of inclusion. (B) Probability of death and MACCE in LC participants. LC, lung cancer; L/F, lost to follow‐up; MACCE, major adverse cardiac and cerebrovascular events.

This is a multicenter, long‐time‐span cohort study utilizing the UK Biobank database. The UK Biobank recruited over 500,000 participants, with an average age at recruitment of 56.5 years (interquartile range (IQR): 50.0 to 63.0) [12]. Participants were recruited from various regions across the United Kingdom, encompassing diverse ethnic backgrounds. The UK Biobank assessed baseline characteristics and tracked participants' primary care records, hospital admissions, cancer records, and mortality records. We obtained data from the UK Biobank online platform in September 2024, with baseline assessment between 2006 and 2010 and follow‐up until October 31, 2022. Out of the 502,173 participants, we excluded those marked as lost to follow‐up (*n* = 1297) and those who had LC prior to recruitment (*n* = 346). Overall, this study included 6095 who were diagnosed with LC over the follow‐up period (Figure 1A). 

### 2.2 Ascertainment of LC and MACCE

The diagnosis of LC was confirmed using ICD‐9 (International Classification of Diseases, 9th Revision) and ICD‐10 (International Classification of Diseases, 10th Revision) codes [13] (Table S3).

We defined MACCE as the outcome, which was a composite outcome encompassing any of the following events: all‐cause mortality, HF, respiratory failure (RF), stroke, shock, and coronary heart disease (CHD). HF, RF, stroke, shock, and CHD were classified using ICD‐10 codes (Table S8). All‐cause mortality was obtained from national death registries. The cut‐off date for updates to the outcomes was October 31, 2022.

### 2.3 Ascertainment of Frailty and Pre‐Frailty

The original conceptualization of the frailty phenotype was articulated and implemented in the Cardiovascular Health Study by Fried and colleagues [14]. This framework has since been adapted for use in the UK Biobank [15, 16]. In this adaptation, weight loss was determined through self‐reporting via the question: “Compared with one year ago, has your weight changed?” with responses coded as “yes, lost weight” = 1 and all other responses = 0. Exhaustion was similarly self‐reported using the question: “Over the past two weeks, how often have you felt tired or had little energy?” Responses indicating “more than half the days or nearly every day” were coded as 1, with all other responses coded as 0. Physical activity was quantified using total metabolic equivalent task (MET) minutes per week, encompassing the sum of walking, moderate, and vigorous activities.

Participants were categorized into quintiles based on sex‐ and age‐specific total MET minutes per week, with the lowest quintile classified as indicative of “low physical activity.”

Gait speed was assessed through self‐report with the question: “How would you describe your usual walking pace?” where ‘slow’ was coded as 1, and all other responses as 0. Hand grip strength was measured using a Jamar J00105 hydraulic hand dynamometer, with results expressed in kilograms adjusted for sex and BMI. Cutoff points for grip strength were aligned with those established by Fried and colleagues [14].

Participants were categorized as frail if they met three or more criteria (weight loss, exhaustion, low physical activity, slow gait speed, weak grip strength), pre‐frail if they met one or two criteria, and non‐frail if they met none.

### 2.4 Ascertainment of Other Covariates

We chose sex, age, ethnic background, family cancer history, smoking status, Townsend deprivation index, obesity, diabetes, hypertension, chronic obstructive pulmonary disease (COPD), and histology classification as they are potential confounders in the frailty and MACCE association or prognostic factors for MACCE among LC participants [17]. Ethnic background is a self‐reported variable, including White, Black and Black mixed, Asian and Asian mixed, and other backgrounds. The study population predominantly consists of individuals with a White ethnic background, while other ethnic backgrounds are more complex and account for a very small proportion of the total population (< 5%). Thus, in Cox regression analysis, we categorized “Black and Black Mixed” and “Asian and Asian Mixed” as “Other”. Body mass index (BMI) was constructed based on height and weight, which were measured during the initial assessment center visit. Some participants' BMI was measured multiple times, and we used the data from the latest visit to the assessment centers. BMI greater than or equal to 30 was defined as obesity. The Townsend deprivation index was used to measure the degree of material deprivation in the population [18]. This index was calculated prior to the recruitment, and each participant is assigned a score corresponding to the output area in which their postcode is located. A larger Townsend deprivation index means a higher degree of material deprivation. The indicator of participants' educational attainment is based on the self‐reported level of academic education and professional skills. If participants provided multiple data points, the most recent data were used. For specific classification methods, please refer to the section on educational attainment classification in Table S1.

Lifestyle‐related indicators include physical activity, smoking status, and alcohol consumption frequency. Regarding physical activity, we used the Summed MET (Metabolic Equivalent Task) minutes, which measure participants' weekly physical activity, covering walking, moderate, and vigorous activities. An average was used if multiple values during follow‐up were provided. Smoking status and alcohol consumption frequency were based on the most recent self‐reported data during participants' visits to the assessment centers.

Tumor histology information was obtained from the cancer registry using the ICD‐O‐3 (International Classification of Diseases for Oncology, 3rd Edition) histology code. For specific tumor classification methods, please refer to the section on tumor histology classification in Table S2. The UK Biobank does not directly provide the stage of LC at initial diagnosis. Tumor stages were assigned based on pathological results, lymph node metastasis, and treatment methods at the time of the initial diagnosis of LC, following the consensus recommendations of the American Cancer Society [19].

### 2.5 Statistical Analysis

Categorical variables were summarized as frequencies and percentages, while continuous variables were expressed as mean ± standard deviation. The normality of continuous data was evaluated using quantile‐quantile plots. In cases where the data deviated from a normal distribution, the interquartile range was reported. To compare means of continuous variables between two groups, *t*‐tests or Wilcoxon rank‐sum tests were employed. For categorical variables, chi‐square tests or Fisher's exact tests were utilized. Comparisons of continuous variables across multiple groups were conducted using ANOVA or Kruskal–Wallis tests, while chi‐square tests or Fisher's exact tests were applied for categorical variables. Cox regression analysis was performed to estimate the hazard ratio of frailty on MACCE, adjusting for sex, age, obesity, family history of cancer, smoking status, Townsend deprivation index, diabetes, hypertension, COPD, tumor type, physical activity, and alcohol consumption. Nearest neighbor propensity score matching (PSM) was performed within each of the study groups. Variables that each cohort was matched included sex, age, family cancer history, smoking status, Townsend deprivation index, obesity, diabetes, hypertension, pathological subtype, and COPD. Continuous variables were rounded to two decimal places, and *p*‐values were rounded to three decimal places. Two‐sided *p*‐values < 0.05 were considered statistically significant. All statistical analyses were conducted using R Project Software (version 4.2.2, The R Foundation).

## 3 Results

### 3.1 Baseline Characteristics

The study included a total of 6095 participants, stratified into three groups based on frailty status: non‐frail (*n* = 2669, 43.79%), pre‐frail (*n* = 2938, 48.20%), and frail (*n* = 488, 8.01%). Table 1 summarizes the baseline characteristics of the study population. The mean age at recruitment was similar across the three groups, with no statistically significant differences observed (non‐frail: 61.50; pre‐frail: 61.46; frail: 61.38, *p* = 0.912). In contrast, the mean age at diagnosis of LC showed significant differences between certain groups. While there was no statistical difference between the non‐frail and pre‐frail groups (69.61 vs. 69.28, *p* = 0.081), the frail group was diagnosed at a significantly younger age compared to the non‐frail group (68.75 vs. 69.61, *p* = 0.007). The frail group had a higher proportion of females compared to the non‐frail group (54.30% vs. 46.87%, *p* = 0.001). White ethnicity predominated across all groups, but the frail group exhibited a slightly higher proportion of participants from Black and Asian ethnicities. Frail participants also had higher rates of obesity (44.88%, *p* < 0.001) compared to the non‐frail and pre‐frail groups. Frail participants were more socioeconomically disadvantaged, as indicated by a higher Townsend deprivation index (*p* < 0.001), and had lower levels of physical activity (*p* < 0.001) compared to pre‐frail and non‐frail participants.

**TABLE 1:** Characteristics of LC participants without frailty, with pre‐frailty, and frailty.

Characteristics	Non‐frail	Pre‐frail	Frail
Total number of patients, *n*	2669	2938	488
Age, y, mean (SD)			
Age at recruitment	61.50 (6.01)	61.46 (5.96)	61.38 (5.58)
Age at diagnosis of LC	69.61 (6.89)	69.28 (7.04)	68.75 (6.35)
Ethnicity, *n* (%)			
White	2595 (97.23%)	2823 (96.09%)	463 (94.88%)
Black or Black mixed background	29 (1.09%)	25 (0.85%)	6 (1.23%)
Asian or Asian mixed background	14 (0.52%)	40 (1.36%)	10 (2.05%)
Other	31 (1.16%)	50 (1.70%)	9 (1.84%)
Sex, *n* (%)			
Male	1418 (53.13%)	1435 (48.84%)	223 (45.70%)
Female	1251 (46.87%)	1503 (51.16%)	265 (54.30%)
BMI, kg/m^2^, *n* (%)			
< 18.5	19 (0.71%)	31 (1.06%)	11 (2.25%)
18.5–24.9	927 (34.73%)	754 (25.66%)	110 (22.54%)
25–29.9	1186 (44.44%)	1224 (41.66%)	144 (29.51%)
≥ 30	509 (19.07%)	907 (30.87%)	219 (44.88%)
Townsend deprivation index, mean (SD)	−0.66 (3.36)	0.04 (3.54)	1.36 (3.56)
Level of education, *n* (%)			
Academic/professional degree	1229 (46.05%)	1179 (40.13%)	142 (29.10%)
Lower degree	1440 (53.95%)	1759 (59.87%)	346 (70.90%)
Lifestyle factors			
Physical activity^a^, mean (SD)	3583.67 (2875.27)	2142.49 (2528.58)	746.61 (1389.75)
Smoking status, *n* (%)			
Non‐smokers	478 (17.91%)	455 (15.49%)	56 (11.48%)
Ex‐smokers	1212 (45.41%)	1300 (44.25%)	184 (37.70%)
Current smokers	951 (35.63%)	1153 (39.24%)	244 (50.00%)
Frequency of alcohol intake, *n* (%)			
Never	216 (8.09%)	359 (12.22%)	95 (19.47%)
Special occasions only	283 (10.60%)	468 (15.93%)	114 (23.36%)
One to three times a month	252 (9.44%)	300 (10.21%)	45 (9.22%)
Once or twice a week	639 (23.94%)	641 (21.82%)	102 (20.90%)
Three or four times a week	560 (20.98%)	510 (17.36%)	67 (13.73%)
Daily or almost daily	709 (26.56%)	646 (21.99%)	65 (13.32%)
Family history of cancer, *n* (%)	1121 (42.00%)	1262 (42.95%)	215 (44.06%)
Diseases before LC diagnosis, *n* (%)			
Diabetes	261 (9.78%)	518 (17.63%)	131 (26.84%)
Hypertension	1122 (42.04%)	1409 (47.96%)	286 (58.61%)
COPD	618 (23.15%)	924 (31.45%)	222 (45.49%)
Histologic subtypes of LC, *n* (%)			
Adenocarcinoma	958 (48.36%)	966 (43.28%)	130 (34.21%)
Squamous cell carcinoma	353 (17.82%)	466 (20.88%)	91 (23.95%)
Large cell carcinoma	22 (1.11%)	20 (0.90%)	3 (0.79%)
Small cell carcinoma	205 (10.35%)	213 (9.54%)	33 (8.68%)
Other specified carcinoma	96 (4.85%)	112 (5.02%)	16 (4.21%)
Unspecified malignant neoplasms	347 (17.52%)	455 (20.39%)	107 (28.16%)
Stage at diagnosis, *n* (%)			
NSCLC			
Stage I–II	435 (17.20%)	493 (17.73%)	67 (14.41%)
Stage III	479 (18.94%)	524 (18.85%)	79 (16.99%)
Stage IV	1410 (55.75%)	1550 (55.76%)	286 (61.51%)
SCLC			
Limited‐stage	11 (0.43%)	9 (0.32%)	2 (0.43%)
Extensive‐stage	194 (7.67%)	204 (7.34%)	31 (6.67%)

Abbreviations: BMI, body mass index; COPD, chronic obstructive pulmonary disease; LC, lung cancer; m, month; METS, metabolic equivalents task score; NSCLC, non‐small cell lung cancer; SD, standard deviation; y, year.

^a^ Physical activity measured by summed metabolic equivalent task minutes per week.

Comorbidities were more prevalent in frail individuals compared to the other groups. Diabetes was present in 26.84% of frail participants, compared to 17.63% in the pre‐frail group and 9.78% in the non‐frail group (*p* < 0.001). Hypertension affected 58.61% of frail individuals, compared to 47.96% in the pre‐frail group and 42.04% in the non‐frail group (*p* < 0.001). COPD was also notably more common in the frail group (45.49%, *p* < 0.001) compared to the pre‐frail and non‐frail groups.

### 3.2 Outcomes

Table 2 outlines the outcomes and cancer‐related treatments among the study population. Frail participants were less likely to undergo surgery (21.93%, *p* = 0.005) and chemotherapy (28.69%, *p* < 0.001) compared to their non‐frail and pre‐frail counterparts. However, the rates of radiotherapy were similar across all groups (approximately 12%). Furthermore, we conducted a series of sensitivity analyses to test the robustness of our findings, including stratified analyses by sex, smoking status, pathological subtype, age, obesity, and excluding newly diagnosed lung cancer patients in the first year (Tables 3, 4, 5, 6, 7, 8).

**TABLE 2:** Clinical treatment and outcome of LC participants without frailty, with pre‐frailty, and frailty.

	Non‐frail	Pre‐frail	Frail	*p*	*p* (non‐ vs. Pre‐frail)	*p* (pre‐ vs. frail)	*p* (non‐ vs. frail)
Total number of patients, *n*	2669	2938	488				
Follow‐up time, d				0.186	0.785	0.073	0.088
Mean (SD)	750.04 (960.12)	757.23 (1006.54)	669.86 (922.43)				
Median (IQR)	349.00 (97.00–1039.00)	328.00 (88.25–1002.50)	301.50 (76.00–825.25)				
Range	0–5095	0–5347	0–4751				
Cancer‐related treatment, *n* (%)							
Surgery	772 (28.92)	831 (28.28)	107 (21.93)	0.005	0.615	0.004	0.001
Chemotherapy	1201 (45.00)	1120 (38.12)	140 (28.69)	< 0.001	< 0.001	< 0.001	< 0.001
Radiotherapy	330 (12.36)	368 (12.53)	60 (12.30)	0.985	0.871	0.941	1
MACCE, *n* (%)	1910 (71.56)	2188 (74.47)	397 (81.35)	< 0.001	0.015	< 0.001	< 0.001
Mortality	1801 (67.48)	2054 (69.91)	377 (77.25)	< 0.001	0.05	< 0.001	< 0.001
HF	101 (3.78)	133 (4.53)	36 (7.38)	0.003	0.181	0.009	< 0.001
CHD	115 (4.31)	163 (5.55)	25 (5.12)	0.099	0.036	0.83	0.404
Stroke	78 (2.92)	87 (2.96)	20 (4.10)	0.347	0.937	0.204	0.2
RF	188 (7.04)	240 (8.17)	45 (9.22)	0.125	0.119	0.426	0.109
Shock	10 (0.37)	12 (0.41)	6 (1.23)	0.052	1	0.033	0.027

Abbreviations: CHD, coronary heart disease; HF, heart failure; IQR, interquartile range; LC, lung cancer; MACCE, major adverse cardiac and cerebrovascular events; RF, respiratory failure; SD, standard deviation; y, year.

**TABLE 3:** Clinical treatment and outcome of LC participants of age more than 65 years old without frailty, with pre‐frailty and frailty.

	Non‐frail	Pre‐frail	Frail	*p* (non‐ vs. pre‐frail)	*p* (pre‐ vs. frail)	*p* (non‐ vs. frail)	*p*
Total number of patients, *n*	1994	2161	347				
Follow‐up time, d				0.398	0.922	0.585	0.664
Mean (SD)	676.49 (864.67)	653.79 (863.58)	648.88 (897.83)				
Median (IQR)	325.50 (89.25–902.00)	295.00 (79.00–887.00)	285.00 (69.00–847.00)				
Range	0–5095	0–5089	0–4751				
Cancer‐related treatment, *n* (%)							
Surgery	582 (29.19)	608 (28.14)	76 (21.90)	0.471	0.016	0.005	0.019
Chemotherapy	836 (41.93)	736 (34.06)	83 (23.92)	< 0.001	< 0.001	< 0.001	< 0.001
Radiotherapy	234 (11.74)	249 (11.52)	39 (11.24)	0.846	0.928	0.856	0.963
MACCE, *n* (%)	1406 (70.51)	1589 (73.53)	276 (79.54)	0.032	0.017	< 0.001	< 0.001
Mortality	1320 (66.20)	1491 (69.00)	257 (74.06)	0.054	0.059	0.004	0.007
HF	88 (4.41)	113 (5.23)	31 (8.93)	0.247	0.009	0.001	0.004
CHD	86 (4.31)	125 (5.78)	19 (5.48)	0.034	0.901	0.326	0.088
Stroke	62 (3.11)	70 (3.24)	16 (4.61)	0.86	0.202	0.147	0.329
RF	150 (7.52)	185 (8.56)	37 (10.66)	0.231	0.221	0.053	0.112
Shock	7 (0.35)	10 (0.46)	5 (1.44)	0.633	0.045	0.022	0.044

Abbreviations: CHD, coronary heart disease; HF, heart failure; IQR, interquartile range; LC, lung cancer; MACCE, major adverse cardiac and cerebrovascular events; RF, respiratory failure; SD, standard deviation; y, year.

**TABLE 4:** Clinical treatment and outcome of LC participants of current smoker without frailty, with pre‐frailty, and frailty.

	Non‐frail	Pre‐frail	Frail	*p* (non‐ vs. pre‐frail)	*p* (pre‐ vs. frail)	*p* (non‐ vs. frail)	*p*
Total number of patients, *n*	951	1153	244				
Follow‐up time, d				0.691	0.104	0.063	0.182
Mean (SD)	665.66 (897.18)	649.95 (903.60)	548.74 (782.71)				
Median (IQR)	301.00 (80.00–786.00)	265.00 (70.00–815.00)	246.00 (62.75–679.75)				
Range	1–4547	0–4822	0–4385				
Cancer‐related treatment, *n* (%)							
Surgery	241 (25.34)	292 (25.33)	45 (18.44)	1	0.026	0.023	0.057
Chemotherapy	412 (43.32)	436 (37.81)	61 (25.00)	0.011	< 0.001	< 0.001	< 0.001
Radiotherapy	112 (11.78)	140 (12.14)	30 (12.30)	0.84	0.915	0.825	0.952
MACCE, *n* (%)	713 (74.97)	922 (79.97)	202 (82.79)	0.007	0.33	0.011	0.004
Mortality	673 (70.77)	868 (75.28)	194 (79.51)	0.02	0.186	0.006	0.007
HF	42 (4.42)	49 (4.25)	12 (4.92)	0.914	0.607	0.73	0.875
CHD	44 (4.63)	62 (5.38)	14 (5.74)	0.484	0.758	0.504	0.64
Stroke	27 (2.84)	38 (3.30)	9 (3.69)	0.613	0.699	0.528	0.702
RF	86 (9.04)	117 (10.15)	27 (11.07)	0.415	0.644	0.328	0.519
Shock	6 (0.63)	3 (0.26)	3 (1.23)	0.315	0.07	0.4	0.101

Abbreviations: CHD, coronary heart disease; HF, heart failure; IQR, interquartile range; LC, lung cancer; MACCE, major adverse cardiac and cerebrovascular events; RF, respiratory failure; SD, standard deviation; y, year.

**TABLE 5:** Clinical treatment and outcome of LC male participants without frailty, with pre‐frailty, and frailty.

	Non‐frail	Pre‐frail	Frail	*p* (non‐ vs. pre‐frail)	*p* (pre‐ vs. frail)	*p* (non‐ vs. frail)	*p*
Total number of patients, *n*	1418	1435	223				
Follow‐up time, d				0.293	0.025	0.068	0.063
Mean (SD)	645.84 (868.91)	681.42 (938.20)	532.36 (817.83)				
Median (IQR)	288.50 (79.00–808.75)	286.00 (79.00–891.50)	226.00 (57.00–686.00)				
Range	0–4722	0–5347	2–4751				
Cancer‐related treatment, *n* (%)							
Surgery	385 (27.15)	353 (24.60)	35 (15.70)	0.124	0.003	< 0.001	< 0.001
Chemotherapy	615 (43.37)	547 (38.12)	52 (23.32)	0.005	< 0.001	< 0.001	< 0.001
Radiotherapy	169 (11.92)	172 (11.99)	28 (12.56)	1	0.825	0.824	0.95
MACCE, *n* (%)	1086 (76.59)	1137 (79.23)	196 (87.89)	0.095	0.002	< 0.001	< 0.001
Mortality	1033 (72.85)	1068 (74.43)	188 (84.30)	0.35	0.001	< 0.001	< 0.001
HF	61 (4.30)	84 (5.85)	16 (7.17)	0.061	0.449	0.086	0.064
CHD	67 (4.72)	90 (6.27)	13 (5.83)	0.071	0.882	0.502	0.18
Stroke	39 (2.75)	47 (3.28)	12 (5.38)	0.444	0.12	0.058	0.124
RF	115 (8.11)	126 (8.78)	21 (9.42)	0.545	0.706	0.514	0.689
Shock	5 (0.35)	5 (0.35)	2 (0.90)	1	0.24	0.244	0.359

Abbreviations: CHD, coronary heart disease; HF, heart failure; IQR, interquartile range; LC, lung cancer; MACCE, major adverse cardiac and cerebrovascular events; RF, respiratory failure; SD, standard deviation; y, year.

Frail participants had the highest incidence of MACCE, with 81.35% experiencing at least one event, compared to 74.47% in the pre‐frail and 71.56% in the non‐frail groups (*p* < 0.001). Frail individuals also demonstrated significantly higher all‐cause mortality (77.25%) compared to pre‐frail (69.91%) and non‐frail participants (67.48%) (Table 9).

**TABLE 6:** Clinical treatment and outcome of LC participants of NSCLC without frailty, with pre‐frailty, and frailty.

	Non‐frail	Pre‐frail	Frail	*p* (non‐ vs. Pre‐frail)	*p* (pre‐ vs. frail)	*p* (non‐ vs. frail)	*p*
Total number of patients, *n*	1776	2019	347				
Follow‐up time, d				0.398	0.08	0.025	0.086
Mean (SD)	882.61 (1029.45)	854.08 (1045.94)	749.15 (942.35)				
Median (IQR)	484.00 (115.75–1265.25)	413.00 (99.00–1190.50)	345.00 (81.00–1000.00)				
Range	1–5038	0–5347	1–4576				
Cancer‐related treatment, *n* (%)							
Surgery	619 (34.85)	636 (31.50)	87 (25.07)	0.029	0.017	< 0.001	< 0.001
Chemotherapy	781 (43.98)	797 (39.47)	97 (27.95)	0.005	< 0.001	< 0.001	< 0.001
Radiotherapy	222 (12.50)	270 (13.37)	40 (11.53)	0.439	0.389	0.656	0.563
MACCE, *n* (%)	1346 (75.79)	1618 (80.14)	291 (83.86)	0.001	0.122	< 0.001	< 0.001
Mortality	1271 (71.57)	1528 (75.68)	281 (80.98)	0.004	0.034	< 0.001	< 0.001
HF	75 (4.22)	99 (4.90)	26 (7.49)	0.351	0.051	0.013	0.039
CHD	83 (4.67)	126 (6.24)	18 (5.19)	0.038	0.543	0.679	0.106
Stroke	57 (3.21)	71 (3.52)	14 (4.03)	0.653	0.639	0.416	0.663
RF	141 (7.94)	170 (8.42)	30 (8.65)	0.594	0.917	0.666	0.816
Shock	9 (0.51)	7 (0.35)	4 (1.15)	0.464	0.064	0.247	0.109

Abbreviations: CHD, coronary heart disease; HF, heart failure; IQR, interquartile range; LC, lung cancer; MACCE, major adverse cardiac and cerebrovascular events; RF, respiratory failure; SD, standard deviation; y, year.

### 3.3 Kaplan–Meier Survival Analysis

**FIGURE 2:**
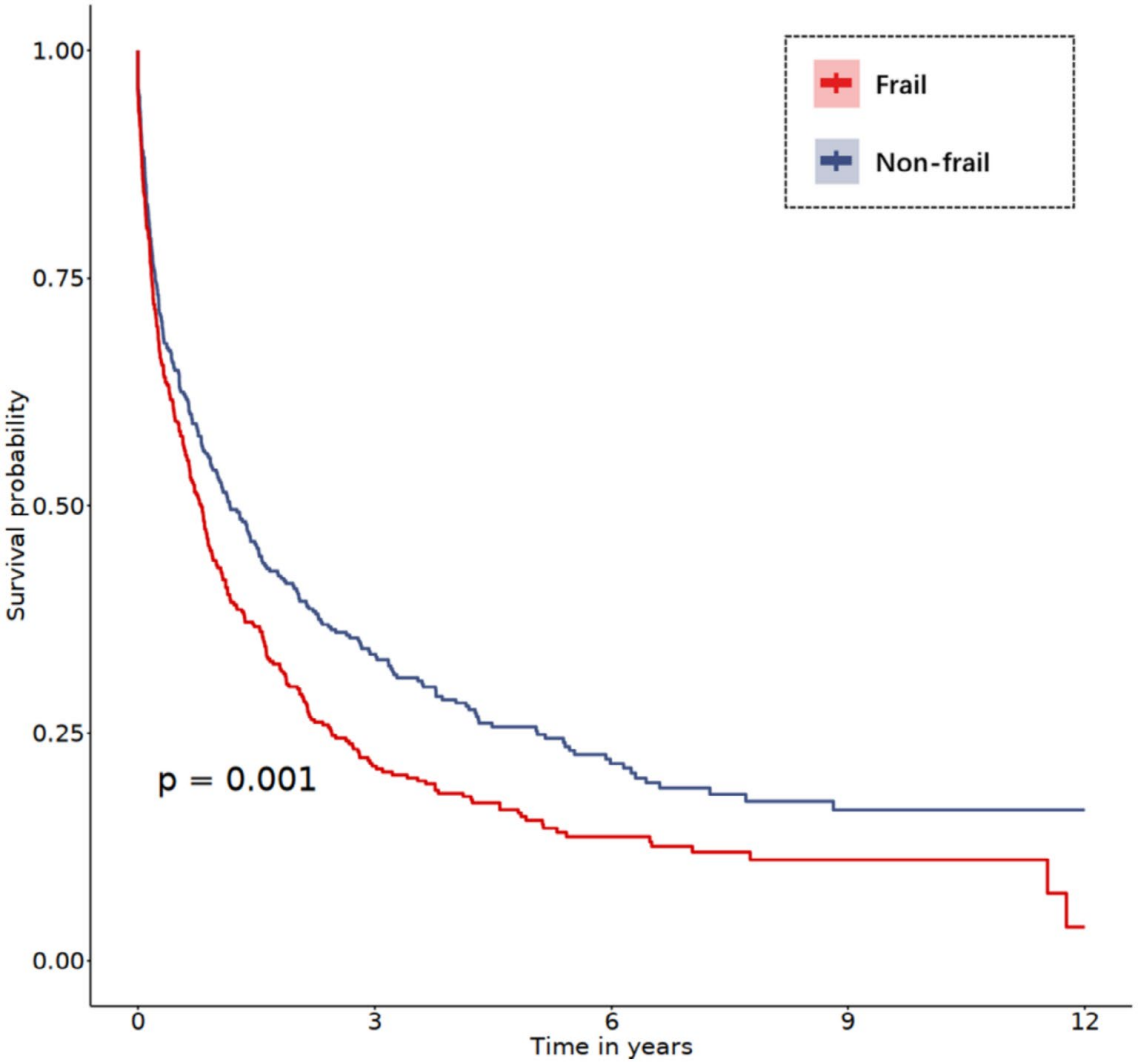
Kaplan–Meier estimates of the probability of MACCE in LC participants without frailty and frailty after PSM.

Figure 1B displays Kaplan–Meier survival curves for mortality and MACCE stratified by frailty status. Frail participants had significantly worse survival outcomes compared to the non‐frail group, with a reduction in survival rates over the 10‐year follow‐up period. For mortality, frail individuals demonstrated significantly lower survival (*p* < 0.001), while pre‐frail individuals also exhibited a trend toward poorer survival, although the difference was not statistically significant (*p* = 0.128). Similarly, frail participants had significantly lower survival from MACCE (*p* < 0.001), while pre‐frail individuals also showed reduced survival compared to non‐frail participants (*p* = 0.019). Furthermore, we compared long‐term MACCE after PSM, and the results showed the frail participants had significantly lower survival from MACCE (*p* = 0.001) after balancing the baseline characteristics across frailty groups (Figure 2).

**TABLE 7:** Clinical treatment and outcome of LC participants with obesity without frailty, with pre‐frailty, and frailty.

	Non‐frail	Pre‐frail	Frail	*p* (non‐ vs. pre‐frail)	*p* (pre‐ vs. FRAIL)	*p* (non‐ vs. frail)	*p*
Total number of patients, *n*	509	907	219				
Follow‐up time, d				0.141	0.85	0.224	0.277
Mean (SD)	808.45 (1008.06)	725.14 (1026.88)	710.70 (962.05)				
Median (IQR)	376.00 (80.00–1120.00)	268.00 (78.00–968.50)	308.00 (79.50–892.50)				
Range	0–4581	0–5347	0–4436				
Cancer‐related treatment, *n* (%)							
Surgery	152 (29.86)	240 (26.46)	56 (25.57)	0.174	0.864	0.247	0.317
Chemotherapy	201 (39.49)	343 (37.82)	68 (31.05)	0.569	0.072	0.036	0.09
Radiotherapy	62 (12.18)	102 (11.25)	21 (9.59)	0.604	0.547	0.374	0.613
MACCE, *n* (%)	349 (68.57)	692 (76.30)	174 (79.45)	0.002	0.372	0.003	0.001
Mortality	328 (64.44)	644 (71.00)	164 (74.89)	0.012	0.277	0.006	0.007
HF	25 (4.91)	60 (6.62)	25 (11.42)	0.243	0.022	0.002	0.008
CHD	26 (5.11)	57 (6.28)	13 (5.94)	0.41	1	0.72	0.676
Stroke	16 (3.14)	25 (2.76)	8 (3.65)	0.742	0.502	0.821	0.705
RF	37 (7.27)	82 (9.04)	20 (9.13)	0.273	1	0.452	0.478
Shock	2 (0.39)	8 (0.88)	2 (0.91)	0.51	1	0.588	0.645

Abbreviations: CHD, coronary heart disease; HF, heart failure; IQR, interquartile range; LC, lung cancer; MACCE, major adverse cardiac and cerebrovascular events; RF, respiratory failure; SD, standard deviation; y, year.

**TABLE 8:** Clinical treatment and outcome of LC participants excluding newly diagnosed lung cancer patients in the first year without frailty, with pre‐frailty and frailty.

	Non‐frail	Pre‐frail	Frail	*p* (non‐ vs. pre‐frail)	*p* (pre‐ vs. frail)	*p* (non‐ vs. Frail)	*p*
Total number of patients, *n*	2567	2813	459				
Follow‐up time, d				0.96	0.166	0.166	0.356
Mean (SD)	744.48 (945.27)	745.81 (975.39)	678.20 (934.75)				
Median (IQR)	346.00 (97.00–1046.00)	328.00 (87.00–1005.00)	301.00 (76.00–831.50)				
Range	0–4829	0–5089	0–4751				
Cancer‐related treatment, *n* (%)							
Surgery	747 (29.10)	799 (28.40)	104 (22.66)	0.587	0.011	0.005	0.016
Chemotherapy	1141 (44.45)	1053 (37.43)	124 (27.02)	< 0.001	< 0.001	< 0.001	< 0.001
Radiotherapy	316 (12.31)	354 (12.58)	56 (12.20)	0.772	0.879	1	0.951
MACCE, *n* (%)	1812 (70.59)	2073 (73.69)	368 (80.17)	0.011	0.003	< 0.001	< 0.001
Mortality	1706 (66.46)	1943 (69.07)	348 (75.82)	0.041	0.004	< 0.001	< 0.001
HF	97 (3.78)	128 (4.55)	33 (7.19)	0.173	0.02	0.002	0.006
CHD	110 (4.29)	155 (5.51)	22 (4.79)	0.043	0.579	0.62	0.116
Stroke	75 (2.92)	87 (3.09)	19 (4.14)	0.75	0.254	0.187	0.371
RF	185 (7.21)	234 (8.32)	42 (9.15)	0.14	0.527	0.149	0.179
Shock	10 (0.39)	11 (0.39)	5 (1.09)	1	0.062	0.064	0.121

Abbreviations: CHD, coronary heart disease; HF, heart failure; IQR, interquartile range; LC, lung cancer; MACCE, major adverse cardiac and cerebrovascular events; RF, respiratory failure; SD, standard deviation; y, year.

**TABLE 9:** Clinical outcome of LC participants without frailty and frailty after propensity score matching analysis.

	Non‐frail (*n* = 373)	Frail (*n* = 373)	*p*
Age at diagnosis of LC, y, mean (SD)	68.28 (7.06)	68.28 (6.21)	0.996
Sex, *n* (%)			1.000
Male	168 (45.04%)	167 (44.77%)	
Female	205 (54.96%)	206 (55.23%)	
Smoking status, *n* (%)			0.909
Non‐smokers	33 (8.85%)	33 (8.85%)	
Ex‐smokers	140 (37.53%)	146 (39.14%)	
Current smokers	200 (53.62%)	194 (52.01%)	
Obesity	140 (37.53%)	158 (42.36%)	0.204
Townsend	0 (0.00%)	2 (0.54%)	0.670
Family cancer history	179 (47.99%)	171 (45.84%)	0.608
Diseases before LC diagnosis, *n* (%)			
Diabetes	80 (21.45%)	92 (24.66%)	0.339
Hypertension	207 (55.50%)	207 (55.50%)	1.000
COPD	174 (46.65%)	177 (47.45%)	0.883
NSCLC	332 (89.01%)	340 (91.15%)	0.391
Outcomes, *n* (%)			
MACCE	285 (76.41%)	317 (84.99%)	0.004
Mortality	263 (70.51%)	307 (82.31%)	< 0.001

Abbreviations: CHD, coronary heart disease; HF, heart failure; IQR, interquartile range; LC, lung cancer; MACCE, major adverse cardiac and cerebrovascular events; RF, respiratory failure; SD, standard deviation; y, year.

### 3.4 Predictors of MACCE

**FIGURE 3:**
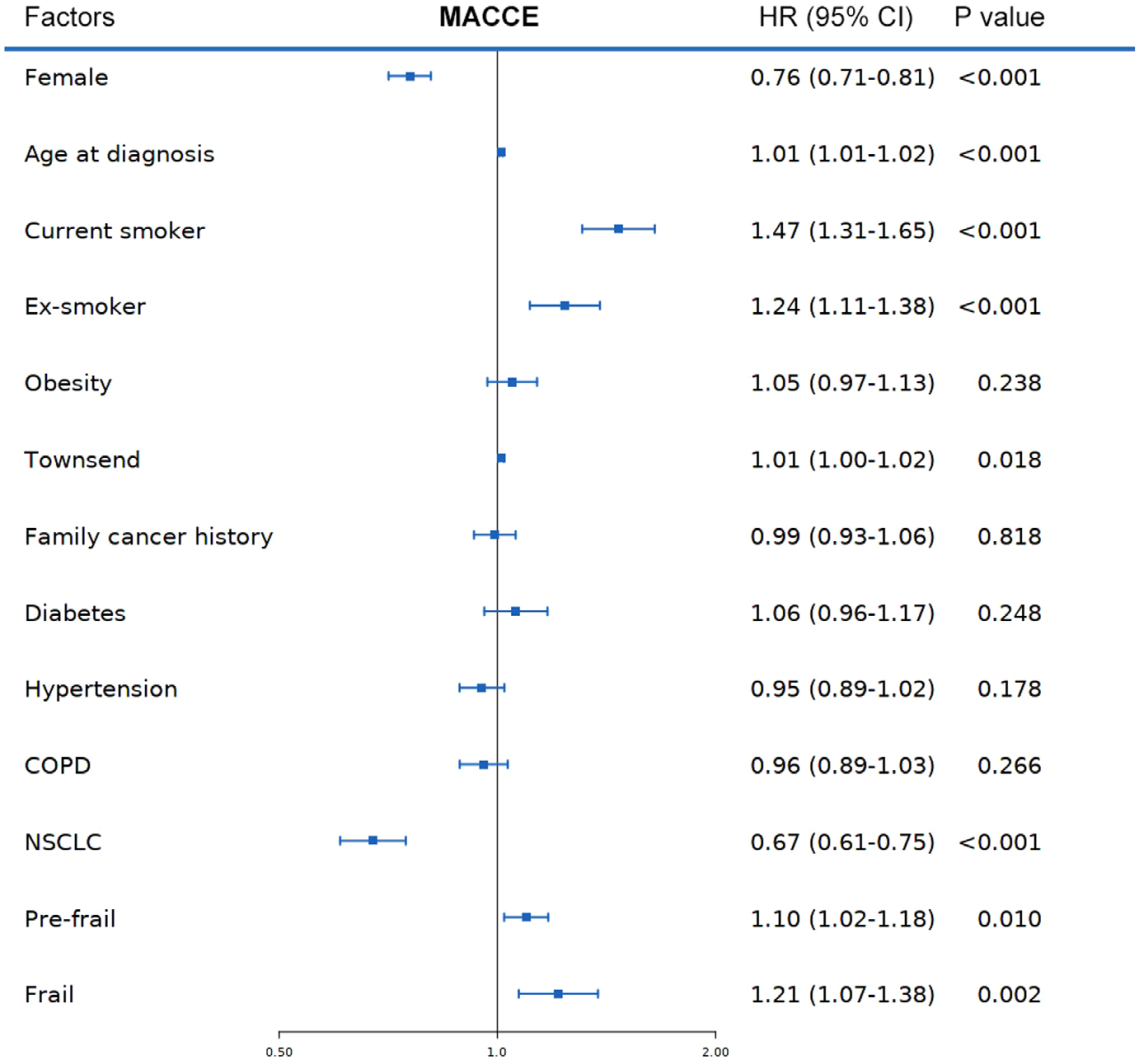
Cox Regression Analysis of LC Patients. Hazard ratio for pre‐frailty, frailty and other covariates. COPD, Chronic obstructive pulmonary disease; HR, hazard ratio; LC, lung cancer; NSCLC, non‐small cell lung cancer.

Cox regression analysis (Figure 3) identified frailty as an independent predictor of MACCE. Being frail was associated with a 21% higher hazard for MACCE (HR = 1.21, 95% CI: 1.07–1.38, *p* = 0.002), while pre‐frailty conferred a 10% increased hazard (HR = 1.10, 95% CI: 1.02–1.18, *p* = 0.010). Smoking status also emerged as a significant predictor, with current smokers (HR = 1.47, 95% CI: 1.31–1.65, *p* < 0.001) and ex‐smokers (HR = 1.24, 95% CI: 1.11–1.38, *p* < 0.001) having higher risks compared to non‐smokers. Female (HR = 0.76, 95% CI: 0.71–0.81, *p* < 0.001) and a diagnosis of NSCLC (HR = 0.67, 95% CI: 0.61–0.75, *p* < 0.001) were associated with lower risks of MACCE.

### 3.5 Frailty Components and Outcomes

**FIGURE 4:**
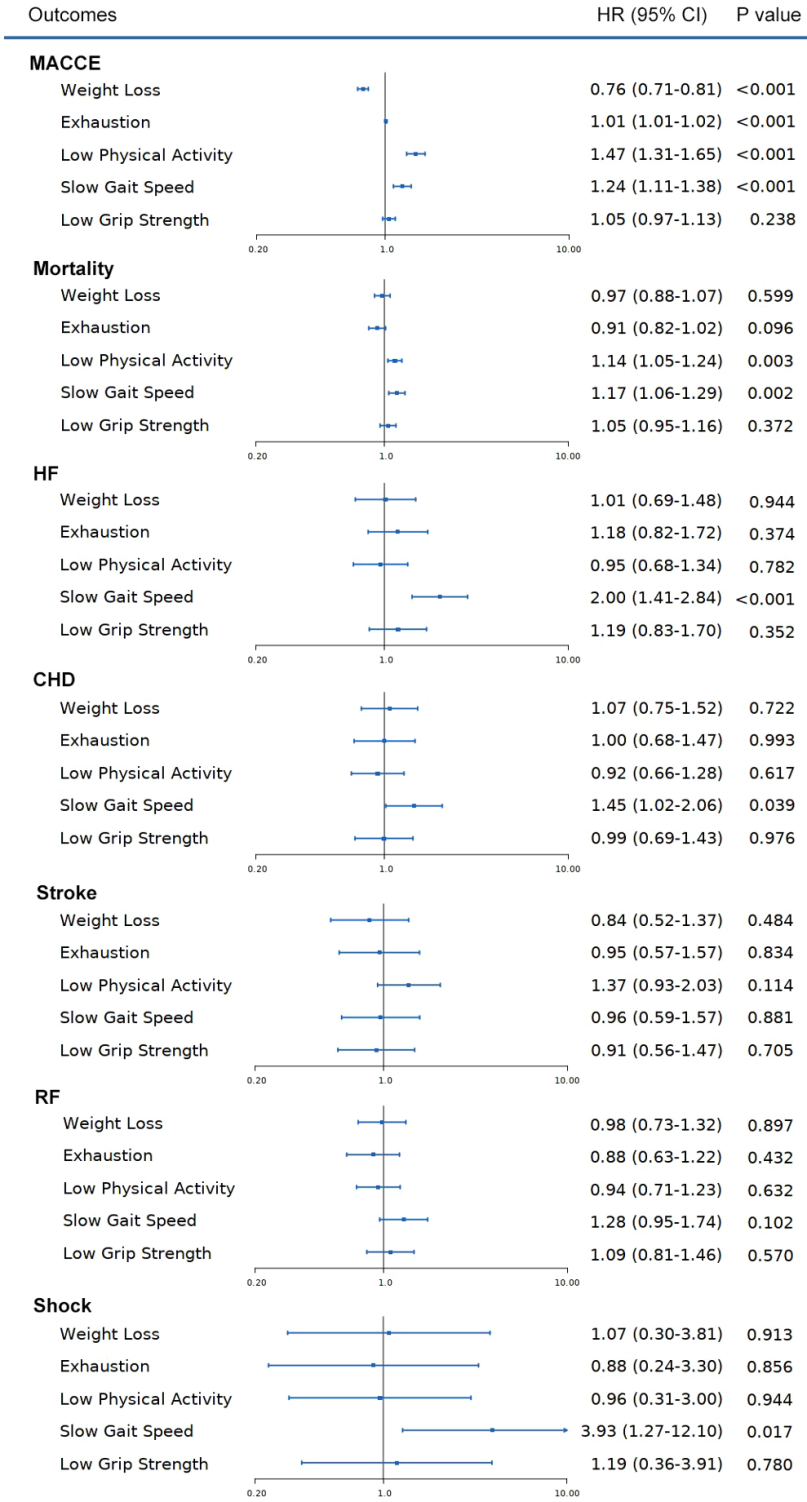
Association between frailty phenotype components and risk of MACCE. Cox proportional hazards models adjusted for age at diagnosis, sex, smoking status, obesity, Townsend deprivation index, family cancer history, diabetes, hypertension, COPD and NSCLC, and mutually adjusted for other frailty components. CHD, coronary heart disease; COPD, Chronic obstructive pulmonary disease; HF, heart failure; HR, hazard ratio; LC, lung cancer; NSCLC, non‐small cell lung cancer; MACCE, major adverse cardiac and cerebrovascular events; RF, respiratory failure.

Figure 4 presents the associations between individual frailty components and various clinical outcomes. Among these components, low physical activity and slow gait speed were consistently associated with increased risks of adverse outcomes. Low physical activity was associated with a 16% higher risk of MACCE (HR = 1.16, 95% CI: 1.05–1.27, *p* = 0.002), a 17% higher risk of mortality (HR = 1.17, 95% CI: 1.06–1.29, *p* = 0.001), and a 61% higher risk of stroke (HR = 1.61, 95% CI: 1.06–2.44, *p* = 0.025). Similarly, slow gait speed was associated with a 68% higher risk of HF (HR = 1.68, 95% CI: 1.12–2.50, *p* = 0.012) and a nearly 5‐fold higher risk of shock (HR = 4.99, 95% CI: 1.32–18.89, *p* = 0.018).

Other frailty components, such as weight loss, exhaustion, and low grip strength, demonstrated weaker or statistically insignificant associations with most outcomes, indicating variability in the prognostic value of these components. Notably, slow gait speed and low physical activity were more strongly associated with cardiovascular, cerebrovascular, and respiratory outcomes compared to other components.

## 4 Discussion

Based on nationwide cohort studies of half a million UK Biobank participants, 6095 participants were diagnosed with LC. The prevalence of pre‐frailty and frailty, based on a widely used Fried frailty phenotype, was 48.20% and 8.01%, respectively. We found that frailty and pre‐frailty were independently associated with an increased risk of long‐term MACCE in LC patients. Specifically, frail patients exhibited a significantly higher risk of MACCE compared to non‐frail participants, while pre‐frail patients also showed an elevated but somewhat lesser risk. Additionally, specific components of frailty, notably low physical activity and slow gait speed, were robust predictors of adverse outcomes, underscoring the multifaceted nature of frailty in this population.

Previous studies have established the association between frailty and poor outcomes in cancer patients [11] particularly in terms of treatment (chemotherapy, radiotherapy, and surgery) tolerance [20, 21, 22] and survival [9, 23, 24, 25] However, the relationship between frailty and long‐term cardiovascular outcomes in LC patients has been less well explored. Our findings demonstrate that both frailty and pre‐frailty are independently associated with an increased risk of MACCE in LC patients. Specifically, frail individuals exhibited a 21% higher hazard (HR = 1.21, 95% CI: 1.07–1.38, *p* = 0.002) and pre‐frail individuals a 10% higher hazard (HR = 1.10, 95% CI: 1.02–1.18, *p* = 0.010) compared to non‐frail counterparts. This association persists even after adjusting for potential confounders, including demographic factors, comorbidities, and lifestyle indicators. The elevated risk of MACCE in frail and pre‐frail patients may be attributable to the cumulative physiological decline and the presence of multiple comorbid conditions, such as diabetes, hypertension, and COPD, which were more prevalent in these groups.

The high prevalence of pre‐frailty (48.20%) observed herein corroborates findings from other cohorts, emphasizing the importance of early identification in at risk populations [26]. The high prevalence of pre‐frailty is particularly noteworthy, as it represents a critical intermediate state that precedes full‐fledged frailty. Pre‐frailty, characterized by the presence of one or two frailty criteria, indicates early physiological decline and increased vulnerability, making it an essential target for early intervention strategies. The identification of nearly half of the LC patient cohort as pre‐frail underscores the imperative need for routine screening and proactive management to prevent progression to frailty, which is associated with more severe adverse outcomes in LC patients.

Among the frailty components assessed, low physical activity and slow gait speed emerged as significant predictors of both MACCE and all‐cause mortality, whereas low grip strength did not demonstrate a significant association. Low physical activity was associated with a 16% higher risk of MACCE (HR = 1.16, 95% CI: 1.05–1.27, *p* = 0.002) and a 17% higher risk of mortality (HR = 1.17, 95% CI: 1.06–1.29, *p* = 0.001). Similarly, slow gait speed was linked to a 68% higher risk of heart failure (HR = 1.68, 95% CI: 1.12–2.50, *p* = 0.012) and a nearly fivefold increase in the risk of shock (HR = 4.99, 95% CI: 1.32–18.89, *p* = 0.018). These findings suggest that certain aspects of physical function are more closely related to cardiovascular and overall health outcomes in LC patients. Low physical activity may contribute to cardiovascular deconditioning and exacerbate underlying heart disease, while slow gait speed may reflect broader systemic frailty and reduced physiological reserve [27, 28, 29]. The lack of significant association with low grip strength may indicate that muscle strength alone is insufficient to capture the multifaceted nature of frailty in the context of LC, where other factors such as endurance and mobility play more critical roles in determining patient outcomes.

### 4.1 Strengths and Limitations

This study has several strengths. Firstly, as the first nationwide, large‐sample‐sized cohort focusing on the long‐term prognosis of LC patients without frailty, with pre‐frailty, and with frailty, the results and conclusions of this study could represent a broader population with better generalizability instead of being limited to clinical settings from retrospective, single‐center, or small‐sample‐sized studies from specialized medical centers. While the large sample size of UK Biobank enhances statistical power, the healthy volunteer bias and limited diversity of its participants may constrain the external validity, and thus the generalizability of our findings should be interpreted with caution. Secondly, owing to the long time span follow‐up, we could evaluate the long‐term prognosis of LC patients and provide important evidence for long‐term cardiovascular outcomes among LC patients without frailty, with pre‐frailty, and with frailty. Thirdly, we utilized baseline characteristics, socioeconomic factors, lifestyle factors, disease history, cancer histologic subtypes, and cancer‐related treatments, aiming to provide a comprehensive and longitudinal perspective on the impact of pre‐frailty and frailty on LC patients by comparing LC participants without frailty, with pre‐frailty, and with frailty.

This study has several limitations. Firstly, due to the long time span of this cohort and rapid development in clinical practice, there will inevitably be heterogeneity in diagnostic and therapeutic approaches for LC and frailty. Secondly, information on socioeconomic level and lifestyle factors (including physical activity, smoking status, and frequency of alcohol intake) was mainly self‐reported. Most of the indicators were measured only once, but the reality is that the values of these indicators may change over time. Thirdly, although the physical activity component was operationalized using self‐reported questionnaires in UK Biobank, which allows large‐scale assessment of habitual activity, it may be less objective than the original Fried criteria that relied on standardized activity questionnaires and direct estimates of energy expenditure. Finally, although we controlled for key individual characteristics and comorbidities, residual confounding was still possible.

## 5 Conclusion

To conclude, LC participants had a high prevalence of pre‐frailty and frailty. The presence of frailty and pre‐frailty significantly increased the risk of MACCE in long‐term LC survivors. Notably, slow gait speed and low physical activity were strongly associated with MACCE compared to other frailty components. The results emphasize the essential need for routine frailty assessments in LC patients to enable effective risk stratification, thereby enhancing both cardiovascular and overall outcomes in this vulnerable population and improving survivorship in lung cancer care.

## Author Contributions

**Fang Zhu:** funding acquisition, writing – original draft, visualization, software, data curation, formal analysis, methodology, validation, investigation, conceptualization. **Qian Zhang:** funding acquisition, writing – original draft, visualization, writing – review and editing, formal analysis, project administration. **Hang Hao:** visualization, data curation, investigation, writing – review and editing. **Wenjie Cai:** formal analysis, writing – review and editing. **Qingquan Luo:** supervision, resources, project administration, writing – review and editing.

## Funding

This study was funded by the National Natural Science Foundation of China (82400483, 82100409).

## Ethics Statement

The UK Biobank has obtained ethics approval from the North West Multi‐Centre Research Ethics Committee (MREC), which covers the entire project. Participants in the UK Biobank provided informed consent at the time of enrollment.

## Conflicts of Interest

The authors declare no conflicts of interest.

## Supporting information


**Table S1:** Education category.
**Table S2:** Neoplasm category.
**Table S3:** Lung and/or bronchus malignant neoplasm.
**Table S4:** Diabetes.
**Table S5:** Hypertension.
**Table S6:** Chronic obstructive pulmonary disease.
**Table S7:** Metastasis.
**Table S8:** Outcome.

## Data Availability

The data that support the findings of this study are available on request from the corresponding author. The data are not publicly available due to privacy or ethical restrictions.

## References

[1] H. Sung , J. Ferlay , R. L. Siegel , et al., “Global Cancer Statistics 2020: GLOBOCAN Estimates of Incidence and Mortality Worldwide for 36 Cancers in 185 Countries,” CA: A Cancer Journal for Clinicians 71, no. 3 (2021): 209–249. 33538338 10.3322/caac.21660

[2] GBD 2019 Diseases and Injuries Collaborators , “Global Burden of 369 Diseases and Injuries in 204 Countries and Territories, 1990–2019: A Systematic Analysis for the Global Burden of Disease Study 2019,” Lancet 396, no. 10258 (2020): 1204–1222. 33069326 10.1016/S0140-6736(20)30925-9 PMC7567026

[3] M. L. Janssen‐Heijnen , R. M. Schipper , P. P. Razenberg , M. A. Crommelin , and J. W. Coebergh , “Prevalence of Co‐Morbidity in Lung Cancer Patients and Its Relationship With Treatment: A Population‐Based Study,” Lung Cancer 21, no. 2 (1998): 105–113. 9829544 10.1016/s0169-5002(98)00039-7

[4] C. L. Shapiro , “Cancer Survivorship,” New England Journal of Medicine 379, no. 25 (2018): 2438–2450. 30575480 10.1056/NEJMra1712502

[5] S. F. Dent , R. Kikuchi , L. Kondapalli , et al., “Optimizing Cardiovascular Health in Patients With Cancer: A Practical Review of Risk Assessment, Monitoring, and Prevention of Cancer Treatment‐Related Cardiovascular Toxicity,” American Society of Clinical Oncology Educational Book 40 (2020): 658. 10.1200/EDBK_286019 32213102

[6] E. Dent , F. C. Martin , H. Bergman , J. Woo , R. Romero‐Ortuno , and J. D. Walston , “Management of Frailty: Opportunities, Challenges, and Future Directions,” Lancet (London, England) 394, no. 10206 (2019): 1376–1386. 31609229 10.1016/S0140-6736(19)31785-4

[7] E. O. Hoogendijk , J. Afilalo , K. E. Ensrud , P. Kowal , G. Onder , and L. P. Fried , “Frailty: Implications for Clinical Practice and Public Health,” Lancet 394, no. 10206 (2019): 1365–1375. 31609228 10.1016/S0140-6736(19)31786-6

[8] A. R. Lyon , T. López‐Fernández , L. S. Couch , et al., “2022 ESC Guidelines on Cardio‐Oncology Developed in Collaboration With the European Hematology Association (EHA), the European Society for Therapeutic Radiology and Oncology (ESTRO) and the International Cardio‐Oncology Society (IC‐OS),” European Heart Journal 43, no. 41 (2022): 4229–4361. 36017568 10.1093/eurheartj/ehac244

[9] K. Komici , L. Bencivenga , N. Navani , et al., “Frailty in Patients With Lung Cancer: A Systematic Review and Meta‐Analysis,” Chest 162, no. 2 (2022): 485–497. 35217002 10.1016/j.chest.2022.02.027

[10] D. H. Kim and K. Rockwood , “Frailty in Older Adults,” New England Journal of Medicine 391, no. 6 (2024): 538–548. 39115063 10.1056/NEJMra2301292 PMC11634188

[11] A. P. Navarrete‐Reyes , A. S. Mateos‐Soria , J. J. Sánchez‐Hernández , and J. P. Negrete‐Najar , “Frailty and Cancer Prognosis,” Current Oncology Reports 26, no. 9 (2024): 991–1020. 38865004 10.1007/s11912-024-01558-x

[12] C. Sudlow , J. Gallacher , N. Allen , et al., “UK Biobank: An Open Access Resource for Identifying the Causes of a Wide Range of Complex Diseases of Middle and Old Age,” PLoS Medicine 12, no. 3 (2015): e1001779. 25826379 10.1371/journal.pmed.1001779 PMC4380465

[13] C. A. Celis‐Morales , P. Welsh , D. M. Lyall , et al., “Associations of Grip Strength With Cardiovascular, Respiratory, and Cancer Outcomes and All Cause Mortality: Prospective Cohort Study of Half a Million UK Biobank Participants,” BMJ (Clinical Research Ed.) 361 (2018): k1651. 10.1136/bmj.k1651 PMC5939721 29739772

[14] L. P. Fried , C. M. Tangen , J. Walston , et al., “Frailty in Older Adults: Evidence for a Phenotype,” Journals of Gerontology. Series A, Biological Sciences and Medical Sciences 56, no. 3 (2001): M146–M156. 11253156 10.1093/gerona/56.3.m146

[15] P. Hanlon , B. I. Nicholl , B. D. Jani , D. Lee , R. McQueenie , and F. S. Mair , “Frailty and Pre‐Frailty in Middle‐Aged and Older Adults and Its Association With Multimorbidity and Mortality: A Prospective Analysis of 493 737 UK Biobank Participants,” Lancet Public Health 3, no. 7 (2018): e323–e332. 29908859 10.1016/S2468-2667(18)30091-4 PMC6028743

[16] F. Petermann‐Rocha , D. M. Lyall , S. R. Gray , et al., “Associations Between Physical Frailty and Dementia Incidence: A Prospective Study From UK Biobank,” Lancet Healthy Longevity 1, no. 2 (2020): e58–e68. 36094146 10.1016/S2666-7568(20)30007-6

[17] S. Zhang , L. Liu , S. Shi , et al., “Bidirectional Association Between Cardiovascular Disease and Lung Cancer in a Prospective Cohort Study,” Journal of Thoracic Oncology 19, no. 1 (2024): 80–93. 37703998 10.1016/j.jtho.2023.09.004

[18] P. Townsend , P. Phillimore , and A. Beattie , Health and Deprivation: Inequality and the North (Routledge, 1988).

[19] J. Niederhuber , J. Armitage , J. Doroshow , et al., Abeloff's ClinicalOncology (2019), 1131.

[20] E. Cavdar , Y. Iriagac , K. Karaboyun , O. Avci , and E. S. Seber , “Prospective Comparison of the Value of CARG, G8, and VES‐13 Toxicity Tools in Predicting Chemotherapy‐Related Toxicity in Older Turkish Patients With Cancer,” Journal of Geriatric Oncology 13, no. 6 (2022): 821–827. 35361561 10.1016/j.jgo.2022.03.004

[21] J. F. Shaw , D. Budiansky , F. Sharif , and D. I. McIsaac , “The Association of Frailty With Outcomes After Cancer Surgery: A Systematic Review and Metaanalysis,” Annals of Surgical Oncology 29, no. 8 (2022): 4690–4704. 35072860 10.1245/s10434-021-11321-2

[22] L. G. Keenan , M. O'Brien , T. Ryan , M. Dunne , and O. McArdle , “Assessment of Older Patients With Cancer: Edmonton Frail Scale (EFS) as a Predictor of Adverse Outcomes in Older Patients Undergoing Radiotherapy,” Journal of Geriatric Oncology 8, no. 3 (2017): 206–210. 28024799 10.1016/j.jgo.2016.12.006

[23] F. Zhang , Y. Yan , and C. Ge , “Prevalence and Impact of Frailty in Pancreatic Cancer: A Systematic Review and Meta‐Analysis Based on 35,191 Patients,” Annals of Surgical Oncology 31, no. 1 (2024): 535–544. 37899415 10.1245/s10434-023-14426-y

[24] D. Boakye , B. Rillmann , V. Walter , L. Jansen , M. Hoffmeister , and H. Brenner , “Impact of Comorbidity and Frailty on Prognosis in Colorectal Cancer Patients: A Systematic Review and Meta‐Analysis,” Cancer Treatment Reviews 64 (2018): 30–39. 29459248 10.1016/j.ctrv.2018.02.003

[25] K. Li , R. Yin , and Z. Li , “Frailty and Long‐Term Survival of Patients With Ovarian Cancer: A Systematic Review and Meta‐Analysis,” Frontiers in Oncology 12 (2022): 1007834. 36324564 10.3389/fonc.2022.1007834 PMC9618815

[26] D. Sezgin , M. O'Donovan , J. Woo , et al., “Early Identification of Frailty: Developing an International Delphi Consensus on Pre‐Frailty,” Archives of Gerontology and Geriatrics 99 (2022): 104586. 34896797 10.1016/j.archger.2021.104586

[27] E. L. Watts , C. E. Matthews , J. R. Freeman , et al., “Association of Leisure Time Physical Activity Types and Risks of All‐Cause, Cardiovascular, and Cancer Mortality Among Older Adults,” JAMA Network Open 5, no. 8 (2022): e2228510. 36001316 10.1001/jamanetworkopen.2022.28510 PMC9403775

[28] Y. Matsuzawa , M. Konishi , E. Akiyama , et al., “Association Between Gait Speed as a Measure of Frailty and Risk of Cardiovascular Events After Myocardial Infarction,” Journal of the American College of Cardiology 61, no. 19 (2013): 1964–1972. 23500222 10.1016/j.jacc.2013.02.020

[29] R. D. Hobbs , E. L. Norton , X. Wu , et al., “Gait Speed Is a Preoperative Indicator of Postoperative Events After Elective Proximal Aortic Surgery,” Journal of Thoracic and Cardiovascular Surgery 163, no. 3 (2022): 886–894.e1. 32684393 10.1016/j.jtcvs.2020.03.165 PMC8722375

