# Supplementary material for: Frailty and Pre‐Frailty in Patients With Lung Cancer and Its Association With Long‐Term MACCE: A Longitudinal Cohort Study
Source: Cancer Med. 2025 Dec 8;14(23):e71458. doi: 10.1002/cam4.71458 (PMC12685562; doi:10.1002/cam4.71458)
Supplement: Supplementary file 1 — Table S1: Education category. Table S2: Neoplasm category. Table S3: Lung and/or bronchus malignant neoplasm. Table S4: Diabetes. Table S5: Hypertension. Table S6: Chronic obstructive pulmonary disease. Table S7: Metastasis. Table S8: Outcome. [file CAM4-14-e71458-s001.docx]

**Supplementary Appendix**

**Table of Contents**

[Table S1: Education Category 2](#_Toc187593372)

[Table S2: Neoplasm Category 3](#_Toc187593373)

[Table S3: Lung and/or Bronchus Malignant Neoplasm 4](#_Toc187593374)

[Table S4: Diabetes 5](#_Toc187593375)

[Table S5: Hypertension 8](#_Toc187593376)

[Table S6: Chronic Obstructive Pulmonary Disease 9](#_Toc187593377)

[Table S7: Metastasis 10](#_Toc187593378)

[Table S8: Outcome 11](#_Toc187593379)

Table S1: Education Category

The data comes from UK Biobank Data-Field 6138 [qualifications].

| Term | Education Status |
| --- | --- |
| College or University degree | Academic or other professional degree |
| NVQ or HND or HNC or equivalent |  |
| Other professional qualifications eg: nursing, teaching |  |
| A levels/AS levels or equivalent | Lower degree |
| O levels/GCSEs or equivalent |  |
| CSEs or equivalent |  |
| None of the above |  |

Table S2: Neoplasm Category

The data comes from UK Biobank Data-Field 40011 [histology of cancer tumor].

| ICD-O-3 Histology Code | Category |
| --- | --- |
| 8015, 8050, 8140, 8141, 8143, 8144, 8145, 8147, 8190, 8201, 8211, 8250, 8251, 8252, 8253, 8254, 8255, 8260, 8290, 8310, 8320, 8323, 8333, 8401, 8440, 8471, 8470, 8480, 8481, 8490, 8503, 8507, 8550, 8570, 8571, 8572, 8574, 8576, 8551 | Adenocarcinoma |
| 8051, 8052, 8070, 8071, 8072, 8073, 8074, 8075, 8076, 8078, 8083, 8084, 8090, 8094, 8120, 8123 | Squamous cell carcinoma |
| 8012, 8013, 8014, 8021, 8034, 8082 | Large cell carcinoma |
| 8002, 8041, 8042, 8043, 8044, 8045 | Small cell carcinoma |
| 8003, 8004, 8022, 8030, 8031, 8032, 8033, 8035, 8200, 8240, 8241, 8243, 8244, 8245, 8246, 8249, 8430, 8525, 8560, 8562, 8575, 8980, 8810, 8890, 8972, 9040, 9133, 8800, 9120, 8802 | Other specified carcinoma |
| 8010, 8011, 8020, 8230, 8046, 8000, 8001, 9590, 9591 | Unspecified malignant neoplasms |

Table S3: Lung and/or Bronchus Malignant Neoplasm

The data for diagnoses (ICD-9) is sourced from UK Biobank Data-Field 41271 [Diagnoses – ICD9]

The data for the first diagnosis date (ICD-9) is sourced from UK Biobank Data-Field 41281 [Date of first in-patient diagnosis - ICD9)

The data for diagnoses (ICD-10) is sourced from UK Biobank Data-Field 41270 [Diagnoses - ICD10]

The data for the first diagnosis date (ICD-10) is sourced from UK Biobank Data-Field 41280 [Date of first in-patient diagnosis – ICD10]

General Practitioner Clinical Event Records are sourced from the UK Biobank Data-Field 42040 [GP clinical event records]. These data are coded using Read v2 and CTV3 (Clinical Term V3) codes.

| Classification | Code |
| --- | --- |
| ICD-9 | 162 Malignant neoplasm of trachea, bronchus and lung  1622 Malignant neoplasm of main bronchus  1623 Malignant neoplasm of upper lobe, bronchus or lung  1624 Malignant neoplasm of middle lobe, bronchus or lung  1625 Malignant neoplasm of lower lobe, bronchus or lung  1628 Malignant neoplasm of trachea, bronchus and lung, other  1629 Malignant neoplasm of bronchus and lung, unspecified |
| ICD-10 | C34 Malignant neoplasm of bronchus and lung  C34.0 Main bronchus  C34.1 Upper lobe, bronchus or lung  C34.2 Middle lobe, bronchus or lung  C34.3 Lower lobe, bronchus or lung  C34.8 Overlapping lesion of bronchus and lung  C34.9 Bronchus or lung, unspecified |
| Read v2 | B22.. B220. B2200 B2201 B220z B221. B2210 B2211 B221z B222. B2220 B2221 B222z B223. B2230 B2231 B223z B224. B2240 B2241 B224z B225. B226. B22y. B22z. |
| CTV3 | X78QS B22z. |

Table S4: Diabetes

The data for diagnoses (ICD-9) is sourced from UK Biobank Data-Field 41271 [Diagnoses – ICD9]

The data for the first diagnosis date (ICD-9) is sourced from UK Biobank Data-Field 41281 [Date of first in-patient diagnosis - ICD9)

The data for diagnoses (ICD-10) is sourced from UK Biobank Data-Field 41270 [Diagnoses - ICD10]

The data for the first diagnosis date (ICD-10) is sourced from UK Biobank Data-Field 41280 [Date of first in-patient diagnosis – ICD10]

| Classification | Code |
| --- | --- |
| ICD-9 | 250 Diabetes mellitus  2500 Diabetes mellitus without mention of complication  2501 Diabetes with ketoacidosis  2502 Diabetes with coma  2503 Diabetes with renal manifestations  2504 Diabetes with ophthalmic manifestations  2505 Diabetes with neurological manifestations  2506 Diabetes with peripheral circulatory disorders  2507 Diabetes with other specified manifestations  2509 Diabetes with unspecified complications  3572 Polyneuropathy in diabetes  25000 Diabetes mellitus without mention of complication (adult-onset type)  25001 Diabetes mellitus without mention of complication (juvenile type)  25009 Diabetes mellitus without mention of compl. (adult/juvenile unspec.)  25010 Diabetes with ketoacidosis (adult-onset type)  25011 Diabetes with ketoacidosis (juvenile type)  25019 Diabetes with ketoacidosis (adult/juvenile unspec.)  25020 Diabetes with coma (adult-onset type)  25021 Diabetes with coma (juvenile type)  25029 Diabetes with coma (unspecified whether adult-onset or juvenile type)  25090 Diabetes with unspecified complications (adult-onset type)  25091 Diabetes with unspecified complications (juvenile type)  25099 Diabetes with unspecified complications (unspecified onset) |
| ICD-10 | E10 Insulin-dependent diabetes mellitus  E10.0 With coma  E10.1 With ketoacidosis  E10.2 With renal complications  E10.3 With ophthalmic complications  E10.4 With neurological complications  E10.5 With peripheral circulatory complications  E10.6 With other specified complications  E10.7 With multiple complications  E10.8 With unspecified complications  E10.9 Without complications  E11 Non-insulin-dependent diabetes mellitus  E11.0 With coma  E11.1 With ketoacidosis  E11.2 With renal complications  E11.3 With ophthalmic complications  E11.4 With neurological complications  E11.5 With peripheral circulatory complications  E11.6 With other specified complications  E11.7 With multiple complications  E11.8 With unspecified complications  E11.9 Without complications  E12 Malnutrition-related diabetes mellitus  E12.0 With coma  E12.1 With ketoacidosis  E12.2 With renal complications  E12.3 With ophthalmic complications  E12.4 With neurological complications  E12.5 With peripheral circulatory complications  E12.6 With other specified complications  E12.7 With multiple complications  E12.8 With unspecified complications  E12.9 Without complications  E13 Other specified diabetes mellitus  E13.0 With coma  E13.1 With ketoacidosis  E13.2 With renal complications  E13.3 With ophthalmic complications  E13.4 With neurological complications  E13.5 With peripheral circulatory complications  E13.6 With other specified complications  E13.7 With multiple complications  E13.8 With unspecified complications  E13.9 Without complications  E14 Unspecified diabetes mellitus  E14.0 With coma  E14.1 With ketoacidosis  E14.2 With renal complications  E14.3 With ophthalmic complications  E14.4 With neurological complications  E14.5 With peripheral circulatory complications  E14.6 With other specified complications  E14.7 With multiple complications  E14.8 With unspecified complications  E14.9 Without complications |

Table S5: Hypertension

The data for diagnoses (ICD-9) is sourced from UK Biobank Data-Field 41271 [Diagnoses – ICD9]

The data for the first diagnosis date (ICD-9) is sourced from UK Biobank Data-Field 41281 [Date of first in-patient diagnosis - ICD9)

The data for diagnoses (ICD-10) is sourced from UK Biobank Data-Field 41270 [Diagnoses - ICD10]

The data for the first diagnosis date (ICD-10) is sourced from UK Biobank Data-Field 41280 [Date of first in-patient diagnosis – ICD10]

| Classification | Code |
| --- | --- |
| ICD-9 | 401 Essential hypertension  405 Secondary hypertension  3482 Benign intracranial hypertension  4010 Essential hypertension, specified as malignant  4011 Essential hypertension, specified as benign  4019 Essential hypertension, not specified as malignant or benign  4050 Secondary hypertension, specified as malignant  4051 Secondary hypertension, specified as benign  4059 Secondary hypertension, not specified as malignant or benign  7962 Elevated blood pressure reading without diagnosis of hypertension |
| ICD-10 | I10 Essential (primary) hypertension  I11 Hypertensive heart disease  I11.0 Hypertensive heart disease with (congestive) heart failure  I11.9 Hypertensive heart disease without (congestive) heart failure  I12 Hypertensive renal disease  I12.0 Hypertensive renal disease with renal failure  I12.9 Hypertensive renal disease without renal failure  I13 Hypertensive heart and renal disease  I13.0 Hypertensive heart and renal disease with (congestive) heart failure  I13.1 Hypertensive heart and renal disease with renal failure  I13.2 Hypertensive heart and renal disease with both (congestive) heart failure and renal failure  I13.9 Hypertensive heart and renal disease, unspecified  I15 Secondary hypertension  I15.0 Renovascular hypertension  I15.1 Hypertension secondary to other renal disorders  I15.2 Hypertension secondary to endocrine disorders  I15.8 Other secondary hypertension  I15.9 Secondary hypertension, unspecified |

Table S6: Chronic Obstructive Pulmonary Disease

The data for diagnoses (ICD-9) is sourced from UK Biobank Data-Field 41271 [Diagnoses – ICD9]

The data for the first diagnosis date (ICD-9) is sourced from UK Biobank Data-Field 41281 [Date of first in-patient diagnosis - ICD9)

The data for diagnoses (ICD-10) is sourced from UK Biobank Data-Field 41270 [Diagnoses - ICD10]

The data for the first diagnosis date (ICD-10) is sourced from UK Biobank Data-Field 41280 [Date of first in-patient diagnosis – ICD10]

| Classification | Code |
| --- | --- |
| ICD-9 | 491 Chronic bronchitis  4910 Simple chronic bronchitis  4911 Mucopurulent chronic bronchitis  4912 Obstructive chronic bronchitis  4918 Other specified chronic bronchitis  4919 Chronic bronchitis, unspecified  492 Emphysema  4929 Emphysema  496 Chronic airways obstruction, not elsewhere classified  4969 Chronic airways obstruction, not elsewhere classified |
| ICD-10 | J41 Simple and mucopurulent chronic bronchitis  J41.0 Simple chronic bronchitis  J41.1 Mucopurulent chronic bronchitis  J41.8 Mixed simple and mucopurulent chronic bronchitis  J42 Unspecified chronic bronchitis  J43 Emphysema  J43.1 Panlobular emphysema  J43.2 Centrilobular emphysema  J43.8 Other emphysema  J43.9 Emphysema, unspecified  J44 Other chronic obstructive pulmonary disease  J44.0 Chronic obstructive pulmonary disease with acute lower respiratory infection  J44.1 Chronic obstructive pulmonary disease with acute exacerbation, unspecified  J44.8 Other specified chronic obstructive pulmonary disease  J44.9 Chronic obstructive pulmonary disease, unspecified |

Table S7: Metastasis

The data for diagnoses (ICD-10) is sourced from UK Biobank Data-Field 41270 [Diagnoses - ICD10]

The data for the first diagnosis date (ICD-10) is sourced from UK Biobank Data-Field 41280 [Date of first in-patient diagnosis – ICD10]

| Metastasis | Code |
| --- | --- |
| Intrathoracic Lymph Node Metastasis | C77.1 Intrathoracic lymph nodes |
| Distant Metastasis | C78 Secondary malignant neoplasm of respiratory and digestive organs  C78.0 Secondary malignant neoplasm of lung  C78.1 Secondary malignant neoplasm of mediastinum  C78.2 Secondary malignant neoplasm of pleura  C78.3 Secondary malignant neoplasm of other and unspecified respiratory organs  C78.4 Secondary malignant neoplasm of small intestine  C78.5 Secondary malignant neoplasm of large intestine and rectum  C78.6 Secondary malignant neoplasm of retroperitoneum and peritoneum  C78.7 Secondary malignant neoplasm of liver  C78.8 Secondary malignant neoplasm of other and unspecified digestive organs  C79 Secondary malignant neoplasm of other sites  C79.0 Secondary malignant neoplasm of kidney and renal pelvis  C79.1 Secondary malignant neoplasm of bladder and other and unspecified urinary organs  C79.2 Secondary malignant neoplasm of skin  C79.3 Secondary malignant neoplasm of brain and cerebral meninges  C79.4 Secondary malignant neoplasm of other and unspecified parts of nervous system  C79.5 Secondary malignant neoplasm of bone and bone marrow  C79.6 Secondary malignant neoplasm of ovary  C79.7 Secondary malignant neoplasm of adrenal gland  C79.8 Secondary malignant neoplasm of other specified sites  C79.9 Secondary malignant neoplasm, unspecified site  C77.0 Lymph nodes of head, face and neck  C77.2 Intra-abdominal lymph nodes  C77.3 Axillary and upper limb lymph nodes  C77.4 Inguinal and lower limb lymph nodes  C77.5 Intrapelvic lymph nodes  C77.8 Lymph nodes of multiple regions  C77.9 Lymph node, unspecified |

Table S8: Outcome

Secondary outcomes: heart failure, coronary heart disease, stroke, respiratory failure, and shock.

The data for diagnoses (ICD-10) is sourced from UK Biobank Data-Field 41270 [Diagnoses - ICD10]

The data for the first diagnosis date (ICD-10) is sourced from UK Biobank Data-Field 41280 [Date of first in-patient diagnosis – ICD10]

| Secondary Outcome | ICD-10 Code |
| --- | --- |
| Heart Failure | I11.0 Hypertensive heart disease with (congestive) heart failure  I11.9 Hypertensive heart disease without (congestive) heart failure  I13.0 Hypertensive heart and renal disease with (congestive) heart failure  I13.2 Hypertensive heart and renal disease with both (congestive) heart failure and renal failure  I50 Heart failure  I50.0 Congestive heart failure  I50.9 Heart failure, unspecified" |
| Coronary Heart Disease | I20 Angina pectoris  I20.0 Unstable angina  I20.1 Angina pectoris with documented spasm  I20.8 Other forms of angina pectoris  I20.9 Angina pectoris, unspecified  I21 Acute myocardial infarction  I21.0 Acute transmural myocardial infarction of anterior wall  I21.1 Acute transmural myocardial infarction of inferior wall  I21.2 Acute transmural myocardial infarction of other sites  I21.3 Acute transmural myocardial infarction of unspecified site  I21.4 Acute subendocardial myocardial infarction  I21.9 Acute myocardial infarction, unspecified  I21.X Presumed acute myicardial infaction (unconfirmed)  I22 Subsequent myocardial infarction  I22.0 Subsequent myocardial infarction of anterior wall  I22.1 Subsequent myocardial infarction of inferior wall  I22.8 Subsequent myocardial infarction of other sites  I22.9 Subsequent myocardial infarction of unspecified site  I23 Certain current complications following acute myocardial infarction  I23.0 Haemopericardium as current complication following acute myocardial infarction  I23.1 Atrial septal defect as current complication following acute myocardial infarction  I23.2 Ventricular septal defect as current complication following acute myocardial infarction  I23.3 Rupture of cardiac wall without haemopericardium as current complication following acute myocardial infarction  I23.4 Rupture of chordae tendineae as current complication following acute myocardial infarction  I23.5 Rupture of papillary muscle as current complication following acute myocardial infarction  I23.6 Thrombosis of atrium, auricular appendage and ventricle as current complications following acute myocardial infarction  I23.8 Other current complications following acute myocardial infarction  I24 Other acute ischaemic heart diseases  I24.0 Coronary thrombosis not resulting in myocardial infarction  I24.1 Dressler's syndrome  I24.8 Other forms of acute ischaemic heart disease  I24.9 Acute ischaemic heart disease, unspecified  I25 Chronic ischaemic heart disease  I25.0 Atherosclerotic cardiovascular disease, so described  I25.1 Atherosclerotic heart disease  I25.2 Old myocardial infarction  I25.3 Aneurysm of heart  I25.4 Coronary artery aneurysm  I25.5 Ischaemic cardiomyopathy  I25.6 Silent myocardial ischaemia  I25.8 Other forms of chronic ischaemic heart disease  I25.9 Chronic ischaemic heart disease, unspecified" |
| Stroke | I60 Subarachnoid haemorrhage  I60.0 Subarachnoid haemorrhage from carotid siphon and bifurcation  I60.1 Subarachnoid haemorrhage from middle cerebral artery  I60.2 Subarachnoid haemorrhage from anterior communicating artery  I60.3 Subarachnoid haemorrhage from posterior communicating artery  I60.4 Subarachnoid haemorrhage from basilar artery  I60.5 Subarachnoid haemorrhage from vertebral artery  I60.6 Subarachnoid haemorrhage from other intracranial arteries  I60.7 Subarachnoid haemorrhage from intracranial artery, unspecified  I60.8 Other subarachnoid haemorrhage  I60.9 Subarachnoid haemorrhage, unspecified  I61 Intracerebral haemorrhage  I61.0 Intracerebral haemorrhage in hemisphere, subcortical  I61.1 Intracerebral haemorrhage in hemisphere, cortical  I61.2 Intracerebral haemorrhage in hemisphere, unspecified  I61.3 Intracerebral haemorrhage in brain stem  I61.4 Intracerebral haemorrhage in cerebellum  I61.5 Intracerebral haemorrhage, intraventricular  I61.6 Intracerebral haemorrhage, multiple localised  I61.8 Other intracerebral haemorrhage  I61.9 Intracerebral haemorrhage, unspecified  I63 Cerebral infarction  I63.0 Cerebral infarction due to thrombosis of precerebral arteries  I63.1 Cerebral infarction due to embolism of precerebral arteries  I63.2 Cerebral infarction due to unspecified occlusion or stenosis of precerebral arteries  I63.3 Cerebral infarction due to thrombosis of cerebral arteries  I63.4 Cerebral infarction due to embolism of cerebral arteries  I63.5 Cerebral infarction due to unspecified occlusion or stenosis of cerebral arteries  I63.6 Cerebral infarction due to cerebral venous thrombosis, nonpyogenic  I63.8 Other cerebral infarction  I63.9 Cerebral infarction, unspecified  I64 Stroke, not specified as haemorrhage or infarction |
| Respiratory Failure | J96 Respiratory failure, not elsewhere classified  J96.0 Acute respiratory failure  J96.00 Acute respiratory failure; Type I [hypoxic]  J96.01 Acute respiratory failure; Type II [hypercapnic]  J96.09 Acute respiratory failure; Type unspecified  J96.1 Chronic respiratory failure  J96.10 Chronic respiratory failure; Type I [hypoxic]  J96.11 Chronic respiratory failure; Type II [hypercapnic]  J96.19 Chronic respiratory failure; Type unspecified  J96.9 Respiratory failure, unspecified  J96.90 Respiratory failure, unspecified; Type I [hypoxic]  J96.91 Respiratory failure unspecified; Type II [hypercapnic]  J96.99 Respiratory failure, unspecified; Type unspecified |
| Shock | R57 Shock, not elsewhere classified  R57.0 Cardiogenic shock  R57.1 Hypovolaemic shock  R57.2 Septic shock  R57.8 Other shock  R57.9 Shock, unspecified |
